# One-dimensional Confinement Effect on the Self-assembly of Symmetric H-shaped Copolymers in a Thin Film

**DOI:** 10.1038/s41598-017-13375-3

**Published:** 2017-10-19

**Authors:** Dan Mu, Jian-quan Li, Sheng-yu Feng

**Affiliations:** 1 Institute of Research on the Structure and Property of Matter, Zaozhuang University, Zaozhuang, 277160 China; 2 Opto-electronic Engineering College, Zaozhuang University, Zaozhuang, 277160 China; 3 School of Chemistry and Chemical Engineering, Shandong University, Jinan, 250100 China

## Abstract

The self-assembly of a reformed symmetric H-shaped copolymer with four hydrophilic branches and one hydrophobic stem was systematically investigated. The existence of vacancies is vital to regulate the sizes of self-assembled cylinders to be able to form a hexagonal arrangement. With the introduction of horizontal-orientated confinement, a micellar structure is formed through a coalescence mechanism. The short acting distance and large influencing area of the confinement produces numerous small-sized micelles. Additionally, the cycled “contraction-expansion” change helps achieve hexagonal arrangement. In contrast, the introduction of lateral-oriented confinement with long acting distance and small influencing area cannot change the cylindrical structure. Under the fission mechanism, in which the larger cylinder splits into smaller ones, it is quite efficient to generate hierarchical-sized cylinders from larger-sized cylinders in the middle region and smaller-sized cylinders near both walls. The results indicate the possibility of regulating the characteristics of a nanomaterial by tuning the molecular structure of the copolymer and the parameters of the introduced confinement, which are closely related to the self-assembly structure.

## Introduction

The application of self-assembly materials in the area of nanotechnology has been developed over the past decades. Among these materials, block copolymers are important candidates in the macromolecular series. Block copolymers have the ability to self-assemble into various structures, which are typically in the range of 10–100 nm^1,2^, and create various shapes with ordered micro-separation, including spheres, cylinders, lamellae and bicontinuous structures. A wide range of high-performance and functional block copolymer materials have been applied for use in electronics^3^, photonics^4^, biomaterials^5^ and so on. To improve the material properties or to achieve the required device functionality, some methodologies have been utilized to control the orientation of block copolymer materials, including the introduction of external fields^6,7^, shear^8,9^, solvent annealing^10^, solvent^11,12^, thermal annealing^13^, and patterned substrates^14–26^. Among these approaches, spatial confinement provides a powerful and efficient method for the fabrication of ordered morphologies, which inspired us to explore the effect and mechanism of confinements with different acting distances and influencing areas on the self-assembly structure.

Two parameters are set to characterize the properties of the confinements: one is the surface properties, and the other is the dimensionality. First, adjusting the interaction between the confinement and components of the block copolymer corresponds to regulating the affinity of the blocks in the copolymer and results in a neutral or non-neutral surface. Therefore, the wetting behavior of the block copolymer can be tuned in a confined environment^27–29^. Additionally, the introduction of the roughness is another efficient method for creating specific surface patterns^9,30^. Second, according to the difference in the shape of the confinement geometry, the confinements are specified as one-dimensional (1D)^28,29,31–35^, two-dimensional (2D)^36,37^, and three-dimensional (3D)^38–41^ confinements and are in the form of two confining walls, concentric rings and spheres, respectively. However, an understanding of the relationship between the confinement conditions and the resulting structures remains a challenge in the field of soft matter^42^.

Compared with 1D confinement, 2D and 3D confinements are more complex. Therefore, the self-assembly mechanism of block copolymers under high-dimensional confinements is more difficult to verify than that induced by 1D confinement. Fortunately, a few clues have been elucidated. Based on the resulting morphologies induced by 2D confinement^43–45^, a 2D-confined structure can be regarded as an integration of a 1D-confined structure via curving up of its structure from two dimensions. Similarly, a 3D-confined structure can be considered the result of the curving structure from three dimensions. To a certain extent, the relationship between 1D, 2D and 3D confinements are analogous to that among a graphite sheet, carbon nanotube and spherical fullerene, respectively. Therefore, 1D confinement is a fundamental element that can be used to deduce the confined morphologies under 2D or 3D confinements. Therefore, in this study, we studied the self-assembly structure of a symmetric block copolymer under 1D confinement by analyzing the density distribution, size, number of the aggregates, and other related data to elucidate the confinement mechanism. The results offer primary information for the explanation of the self-assembly mechanism, contribute to the deduction of the resulting structures of the block copolymers under 2D and 3D confinements, and furthermore provide reliable theoretical evidence for the preparation of ordered materials.

## Coarse-grained Model and Parameter Settings

The construction of the poly(ethylene oxide) (PEO) and poly(methyl methacrylate) (PMMA) coarse-grained model from an atomic model was described in detailed in our previous study^46^. A brief description is provided as follows. Three parallel models for both the PEO and PMMA homopolymers with various chain lengths were constructed, and calculations of the solubility parameters were performed via molecular simulations. Based on the change in the solubility parameter depending on the chain length in the PEO and PMMA homopolymer models, the representative chain lengths for both PEO and PMMA were determined to be 50, and the corresponding solubility parameters were the threshold for the stable solubility parameters. After dividing by the characteristic ratio, the mesoscopic structures of the shortest EO and MMA segments were constructed by five A beads and six B beads, respectively. Therefore, a symmetric H-shaped copolymer consists of one MMA segment located in the center and four EO segments with the same chain length connecting pairwise to the two end points of the MMA segment.

To run the mesoscopic simulation, we employed MesoDyn, which applies dynamic mean-field density functional theory (DDFT) and was developed by Fraaije^47,48^. The phase-separation dynamics and ordering processes of the polymeric materials are described by Langevin equations. The primary advantage of this method is that there are no a priori assumptions for the phases or phase formation kinetics^49^. The MesoDyn method has been successfully employed to study phase separation^50,51^, phase transition^52^ and self-assembly structures resulting from nanoparticle doping^53,54^, or induced by surfaces^8,30^. Though pioneering and early studies of block copolymers in thin films were done by G. J. A. Sevink *et al*.^55–60^, in this paper, we investigate the structure and corresponding mechanism of a kind of symmetric-structured block copolymer consisting of four hydrophilic branches and one hydrophobic branch undergoing self-assembly in a thin film, with particular focus on the induced structure under a confined environment. Our results enhance previous knowledge on this subject.

The mesoscopic simulations were performed using a *L*_*x*_ × *L*_*y*_ × *L*_*z*_ spatial lattice. To ensure the isotropy of all grid-restricted operators, the bond length was set to 1.1543 Å. For simplicity, all the beads were set to have the same size, and the bead diffusion coefficients were taken to be 1.0 × 10^−7^ cm^2^ ⋅ s^−1^. In addition, the constant noise-scaling parameter was chosen to be 100, the compressibility parameter was fixed at 10.0, and the dimensionless time step was 0.5. Furthermore, the resulting Gaussian chain density functions consisted of a matched relationship between the external potential fields and the density fields for each bead type. The chemical potentials are the independent variables of the external potential and the density field function. The relationship between the time derivatives and the chemical potentials are represented by coupled Langevin equations. The total simulation time for each simulation was 500 × 10^3^ steps.

The pair-wise interactions of the component beads were defined by their Flory-Huggins interaction parameters, *χ*, after multiplication by *RT* (where *R* is the molar gas constant and *T* is the ambient temperature, 400 K), and the resulting physical quantity was *ε*_AB_, which was used as the interaction parameter in the mesoscopic simulations. The *ε*_AB_ value in this work was 6.23 kJ ⋅ mol^−1^, which was obtained from our previous study^28,29^. Here, a positive *ε*_AB_ value corresponds to a net repulsive interaction between the A and B beads, which promotes microphase separation or even phase separation. The adopted 1D confinements were neutral-wall-type confinements with non-preferential wetting for both polymeric components, and the interactions of the wall with both the A and B beads (*ε*_wall−A_ and *ε*_wall−B_) were the same (i.e., 5.0 kJ ⋅ mol^−1^). However, the placement of the walls in the system is different, one is placed in the XY plane, which is referred to as ZW55 confinement, while the other is placed in the XZ plane, which is referred to as YW55 confinement. To study the induced self-assembly structure under ZW55 confinement, mesoscopic simulations were performed in a 100 × 100 × 5 nm^3^ grid to model a thin film by applying a short acting distance (i.e., 5 nm) and large influencing area (i.e., 10,000 nm^2^). Additionally, a 50 × 50 × 5 nm^3^ grid was taken to study the effect of YW55 confinement on the self-assembly structure, whose relatively short acting distance (i.e., 50 nm) is quite fit to make the copolymer molecules between the two walls sensitive to the interaction of the confined walls; in contrast, the 100 nm acting distance is too long, and therefore, the 100 × 100 × 5 nm^3^ grid is not used in the study of YW55 confinement.

## Results and Discussion

### Copolymer in Thin Film

The Z axis dimension is 5 nm, which is 5% of both the X- and Y-axis dimensions, and is therefore proper to apply as the frame of the thin film. The copolymer molecules self-assemble into cylindrical core-shell structures, in which the B and A beads aggregate into the core and shell, respectively. One point on the axis of a cylinder (parallel to the Z axis) was randomly selected as the starting point, and then, the density distributions were drawn along different directions in the same XY plane. To enrich the samples and results, an extra five points on the same cylinder axis were randomly employed as five additional starting points, and the density distributions in the respective XY planes were also produced. All the density distributions nearly overlap, and nearly the same density values for the B beads are found along the same cylinder axis; therefore, the cylinder structure is confirmed. Owing to the high symmetry of the copolymer molecules, a symmetric core-shell structure is formed in the XY plane, in which the hydrophobic B beads are the core and the hydrophilic A beads are the shell. Then, all the similar 2D core-shell sheets assemble together layer by layer under the driving force of the aggregation of the same components, forming a 3D core-shell-structured cylinder.

**Figure 1:**
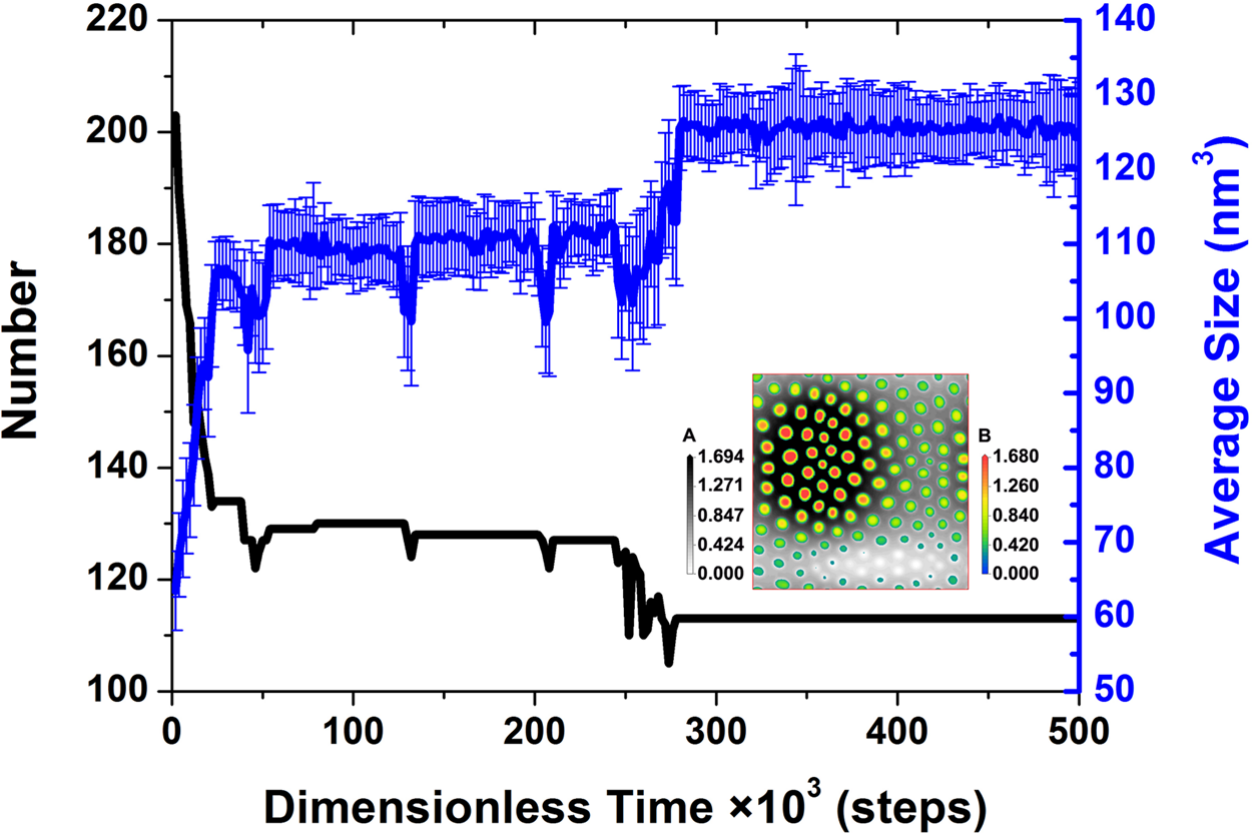
Time evolution of the number (in black) and average size (in blue) of aggregates. Each error bar was calculated from the sizes of all aggregates generated at each representative time. The inset is the density slice in the XY plane of both the A bead (with a grayscale legend) and B bead (with a reverse rainbow legend) at 254 × 10^3^ steps.

When we focus on the whole system, the self-assembly mechanism can be much more clearly explored, and the evolution of the number and average size of the cylinders in the system over time is shown in Fig. 1. Many small aggregates are generated in the early stage before the first 24 × 10^3^ steps, and then, the aggregates enter the arrangement adjustment phase for the following 14 × 10^3^ steps. At the later stage from 38 × 10^3^ steps to 244 × 10^3^ steps, both the number and average size essentially do not vary. Thus, there are three identical “reduction-increase-constant” stages. One or two vacancies arise during three “reduction-increase” stages, which accelerate the arrangement of the aggregates, and thus, the time for three stages to complete reduces, indicating the approach to the final arrangement. However, an opposite trend in the number and average size occurs from 244 × 10^3^ steps to 278 × 10 ^3^ steps; from the inset of the representative density slice at 254 × 10^3^ steps, we can see that more vacancies appear in the small-aggregate region, which facilitate the coalescence of small aggregates. Therefore, the number of aggregates is stabilized to 113 after 278 × 10^3^ steps, and accordingly, the average size is constant at approximately 126 nm^3^. Though the uniformity in the size of the aggregates generated at the same time is not high, a hexagonal arrangement of cylinders is finally formed. Thus, it can be seen that the generation of vacancies is quite vital during the self-assembly of the thin film.

**Figure 2:**
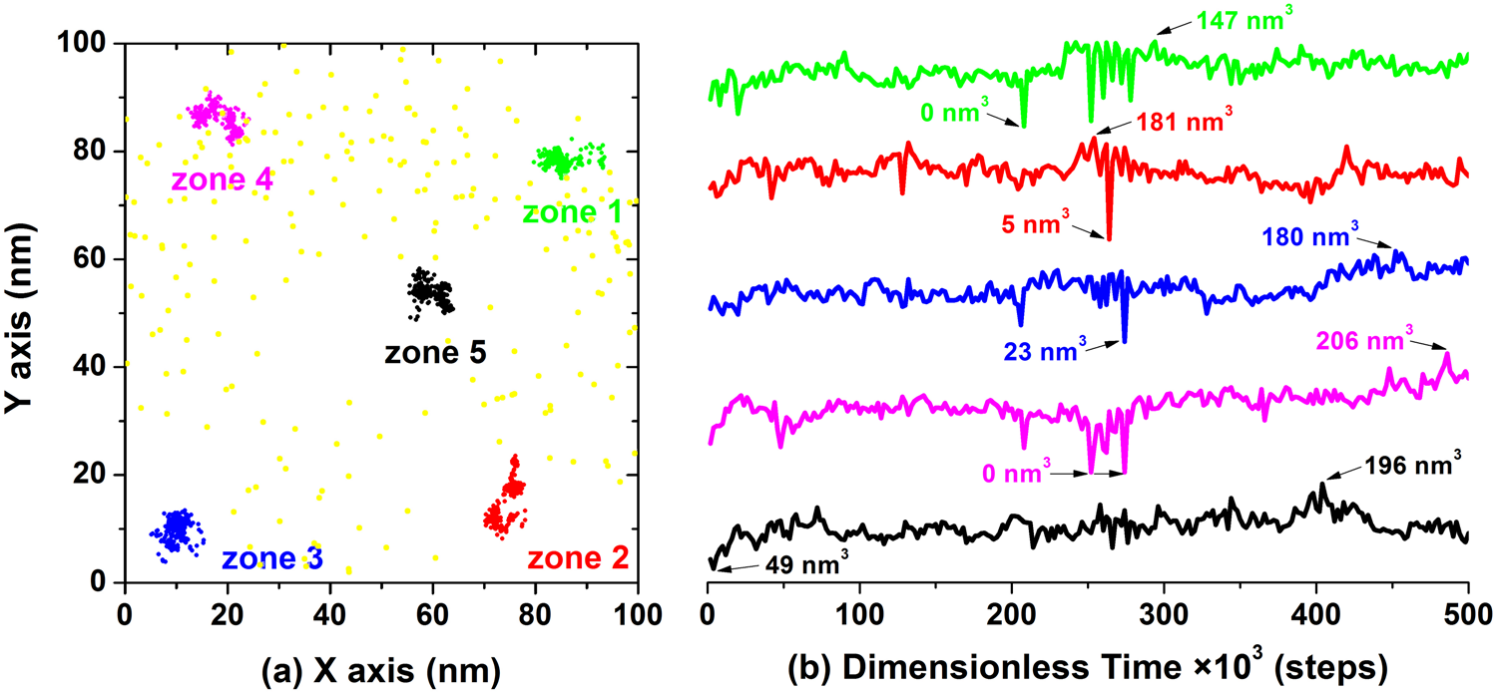
The statistical information of the aggregates in the thin film. (**a**) The positions at which five individual aggregates appear in the selected zones over time in the XY plane, denoted by magenta, green, red, blue and black points. The yellow points indicate the positions of the appearance of vacancies during the whole simulation time. (**b**) Time evolution of the corresponding sizes of the five individual aggregates. For clarity, both the minimum and maximum values of the aggregate sizes are labelled below and above the individual line in the same color as the corresponding line.

To clarify the positions of the vacancies that appeared, the centers of vacancies are denoted by yellow points in Fig. 2(a). From the distribution of all the points, vacancies are not generated at the four corners (i.e., zone 1–4) or the center zone (i.e., zone 5), which indicates the seldom appearance of small aggregates in these five zones. Therefore, five individual aggregates were randomly selected in these five zones, and their positions were tracked. Furthermore, the time evolution of the volume of individual aggregates was also recorded, as shown in Fig. 2(b). The difference between the volume change in zone 1–4 and zone 5 is quite obvious, and all significant fluctuations occur between 244 × 10^3^ steps and 278 × 10^3^ steps, in which the former coincides with the time at which numerous vacancies are formed, but there is no such region of variation for the latter. Except for the vacancies that appear after 208 × 10^3^ steps, all the minimum volumes also appear during this time span. All this proves that zones 1–4 are the active variation zones during the self-assembly process at which vacancies appear and small aggregates coalesce. However, compared with zones 1–4, the occupied moving range and the variation in size are both the smallest in zone 5, and no vacancies are generated in zone 5, proving that the middle region including zone 5 is the relatively inactive during the self-assembly process.

### ZW55 Confinement Effect Mechanism

Placing two parallel walls at the top and bottom of the system in the XY plane forms the ZW55 confined thin film. To demonstrate the self-assembly structure of an individual aggregate, a density analysis was carried out on a randomly selected aggregate. The starting point was set at the geometric center of this aggregate, and the probe of the density of the B beads began at the starting point and moved along the three principal axes separately. The three nearly overlapped density distributions indicate a micellar structure, which is different from the cylindrical structure of the unconfined thin film, resulting from the repulsive interaction of the confinement with the components of the copolymer molecules. The molecular orientation of the copolymer molecules is strongly influenced by the ZW55 confinement due to the synergistic effect of the short acting distance of 5 nm and the large influencing area of 10,000 nm^3^, which explains why the self-assembly structure is completely different. The copolymer molecules in the middle region between the two walls (i.e., far away from the two walls) adopt an approximately parallel orientation to the walls, while the aggregation of the B beads forms the hydrophobic core and the aggregation of the A beads forms the hydrophilic shell in nearly planar area. Due to the influence of the repulsive force of both walls on both the A and B beads, as the copolymer molecules approach the walls, the inclination angle of the molecules to the wall increases. Owing to the highly symmetric H-shaped structure of the copolymer and the symmetrical force from the opposite walls, the inclination angles of each copolymer molecule to both walls are nearly the same. All the copolymer molecules with various inclination angles to the walls aggregate with the same component, and a spherical aggregation, i.e., a micelle, is generated.

**Figure 3:**
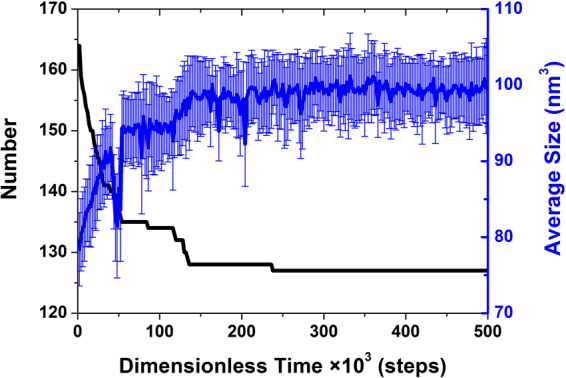
Time evolution of the number (in black) and average size (in blue) of aggregates. Each error bar was calculated from the sizes of all aggregates generated at each representative time.

Different from the three “reduction-increase-constant” stages of the average size in the thin film, a “reduction” in the early period combined with the three “constant-reduction” stages in the later period constitute a new evolutionary trend in the average size under ZW55 confinement, as shown in Fig. 3. At the beginning, compared with the copolymer molecules in the thin film, the repulsive force from the walls speeds the aggregation of the same component, and thus, fewer aggregates (164) with larger average sizes of 78 nm^3^ are generated. With the coalescence of small aggregates into larger ones, the number of aggregates decreases, and the average size increases until reaching 44 × 10^3^ steps, which indicates a fast coalescence mechanism. Afterwards, three vacancies appear one by one at 46, 48 and 52 × 10^3^ steps. Owing to the shrinkage of the micellar size around the vacancies, the average size dropped abruptly, which is also a sign of entering a new period. Starting at 54 × 10^3^ steps, the three primary “constant-reduction” stages of the number of aggregates occur, and the coalescence period is completed after 182 × 10^3^ steps, which indicates a slow coalescence mechanism. Except for the coalescence of six small micelles into larger ones in the number reduction stage from 116 × 10^3^ steps to 136 × 10^3^ steps, only one small micelle fuses into a larger micelle, so the corresponding change in the average size is not obvious. At 238 × 10^3^ steps, the number of micelles remains constant at 127, which is 14 more than in the thin film, and accordingly, the average size also reaches a relative constant of approximately 100 nm^3^. In the last 262 × 10^3^ steps, the self-assembled micelles adjust their position and size over a small range, until achieving a more perfect hexagonal arrangement. Therefore, ZW55 confinement is suitable for producing larger-number and smaller-sized micelles.

**Figure 4:**
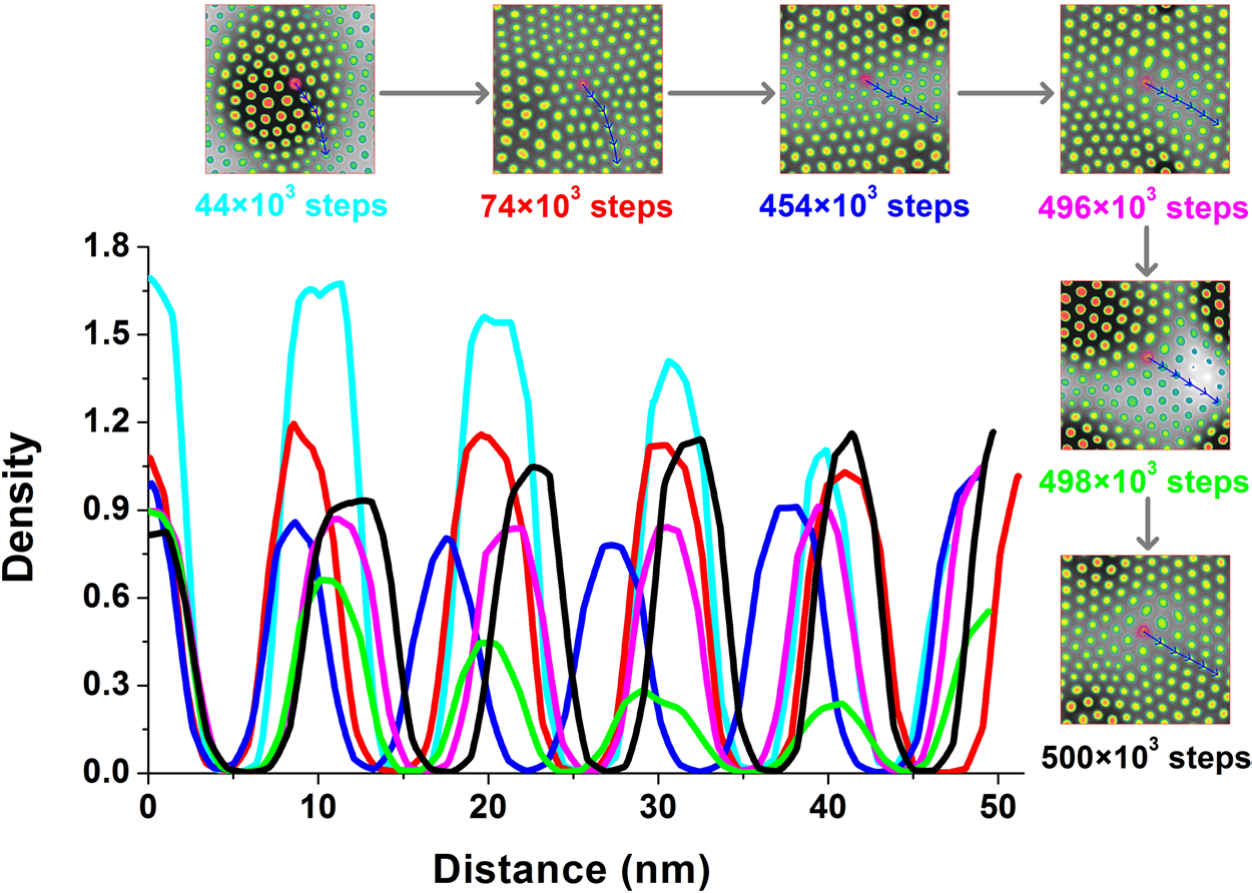
Density distribution of B beads beginning from the aggregate colored in magenta along the same selected route (labelled in blue with arrows in the density slices) at different times, i.e., 44 × 10^3^ steps (in cyan), 74 × 10^3^ steps (in red), 454 × 10^3^ steps (in blue), 496 × 10^3^ steps (in magenta), 498 × 10^3^ steps (in green) and 500 × 10^3^ steps (in black). The density slices are displayed at both the top and right sides according to the time sequence.

**Figure 5:**
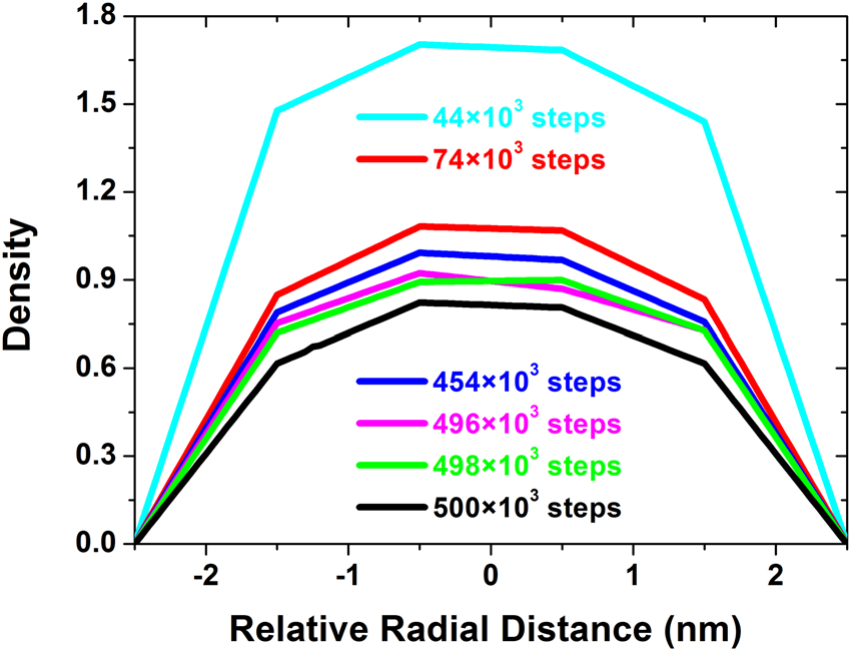
Density distribution of the starting micelle along the maximum radial direction in the Z axis at different times, i.e., 44 × 10^3^ steps (in cyan), 74 × 10^3^ steps (in red), 454 × 10^3^ steps (in blue), 496 × 10^3^ steps (in magenta), 498 × 10^3^ steps (in green) and 500 × 10^3^ steps (in black).

To illustrate the evolution of the hexagonal arrangement, the center of a micelle in the middle region was randomly selected as the starting point, and a probe of the density of the B beads was moved outwards through the other five micelles in the same route, as shown in Fig. 4. At 44 × 10^3^ steps, along the probe direction, the density change in the micellar cores decreases by nearly the same amount, and the diameters of the micelles also show a sharp decreasing trend, as shown in Table 1. The whole system presents a tendency to contract inward, and thus, this is considered to be the accumulating stage of aggregation. After 30 × 10^3^ steps, assuming the arc-shaped route remains unchanged, redistribution of the copolymer molecules leads to a similar density and growth of each micelle. Then, the whole system tends to expand outward, which is the beginning of the adjustment of both the position and size of micelles. At 454 × 10^3^ steps, the average micellar diameter reaches a maximum, with a decrease in the density. The whole system contracts inward slightly, which aligns all the micelles along this route. The density of an individual micelle remains unchanged, but an obvious change in position is observed to optimize the arrangement during the following 42 × 10^3^ steps.Due to the density perturbation, the fine tuning of the arrangement is completed at 500 × 10^3^ steps. Over time, except for the error of the decreasing diameter of six micelles, the uniformity of the micellar size increases, and the density change verifies these results, as shown in Fig. 5. The dramatically high density at the early stage (44 × 10^3^ steps) indicates that the copolymer molecules are concentrated in the middle region, which allows the copolymer to undergo the following redistribution. As time passes, the gradually decreasing densities cause the convergence of the individual micellar density, accompanied by adjustments in both the micellar size and arrangement.

**Table 1:** The individual diameter in nm of micelles along the same route at various times in Fig. 4.

Time (steps)	Diameter 1	Diameter 2	Diameter 3	Diameter 4	Diameter 5	Diameter 6	Average (nm)	Error
44 × 10^3^	8.50	9.05	9.05	9.00	7.20	5.90	8.12	1.30
74 × 10^3^	8.40	9.15	9.70	8.65	9.00	6.90	8.63	0.96
454 × 10^3^	7.90	8.45	8.65	9.45	9.60	9.90	8.99	0.78
496 × 10^3^	9.80	9.50	8.75	8.10	8.75	9.00	8.98	0.60
498 × 10^3^	9.50	8.60	8.05	9.10	8.00	8.90	8.69	0.59
500 × 10^3^	9.50	9.20	8.70	8.95	7.95	7.90	8.70	0.66

### YW55 Confinement Effect Mechanism

**Figure 6:**
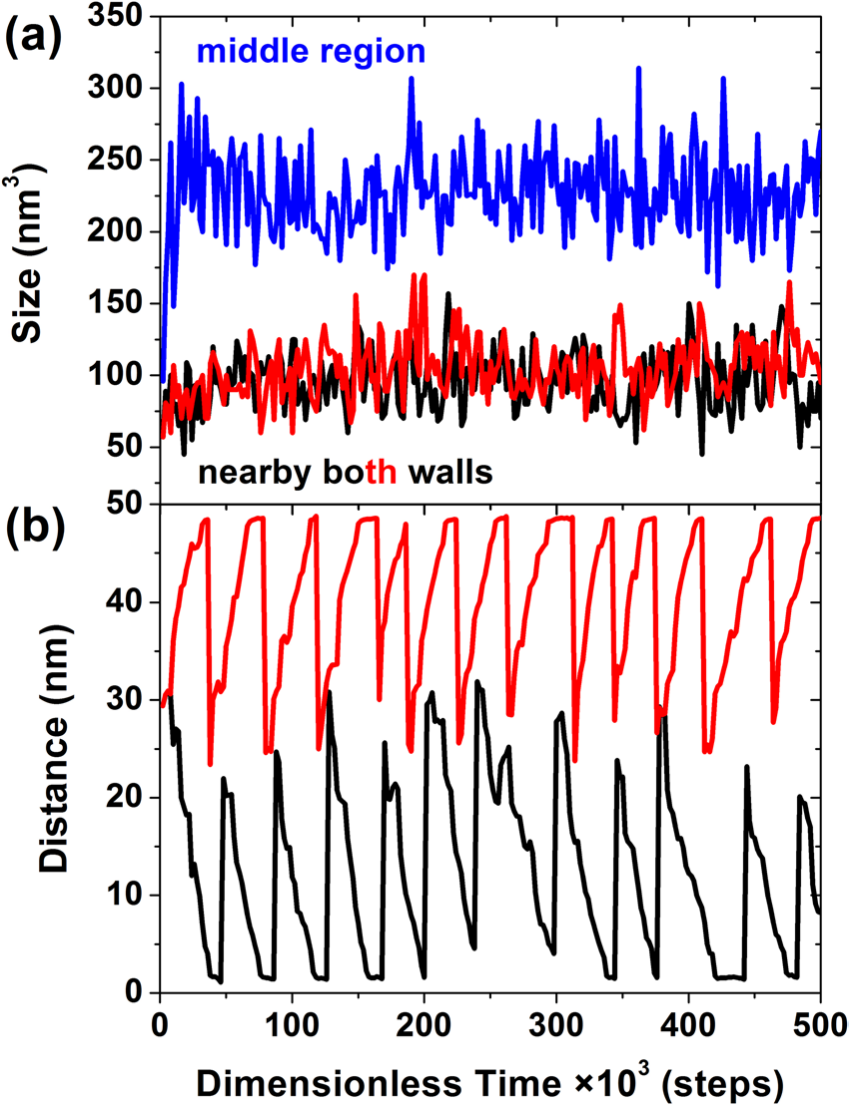
(**a**) Time evolution of the size of the selected aggregates from the middle region (in blue) and from the regions close to two walls (in black and red). (**b**) Time evolution of the distance between two selected aggregates and two adjacent walls, which are denoted by red and black lines.

Compared with ZW55 confinement, YW55 confinement possesses a longer acting distance (i.e., 50 nm) and smaller influencing area (i.e., 250 nm^2^), which is realized by placing both walls in the XZ planes at the edges of the system. The weak repulsive force from the two walls makes the effect of YW55 confinement on the self-assembly structure quite limited. Different from the micellar structure formed under ZW55 confinement, a deformed cylindrical structure self-assembled under YW55 confinement. Owing to the special location of the walls, the copolymer molecules near the two walls suffer the most repulsive force, whereas the molecules in the middle region are not sensitive to the repulsive force from the walls. Therefore, the cylindrical sizes in the whole system are not similar or uniform, and instead present a hierarchical transformation in size under the hierarchical transformation of the force from the opposite walls, as shown in Fig. 6(a). The sizes of the cylinders near the two walls are quite similar, but different from the size of the cylinder in the middle region. Moreover, the former sizes are both smaller than the latter size. The intense repulsive force from the walls on the self-assembled cylinders near the walls leads to the shrinkage of both the EO and MMA segments; therefore, there are only smaller-sized cylinders located near both walls and no larger-sized ones. Conversely, because the distance from the copolymer molecules to both walls is nearly equal, the repulsive force from two walls is similar. The opposite direction, i.e., the offset, of the repulsive forces gives rise to a small resultant force, which helps the self-assembled segments adopt an expanding state, inducing a larger size. When the position of a cylinder in the middle region is tracked, as shown in Fig. 6(b), the same movement direction of starting from the middle region and ending near both walls is detected, and the movement occurs in cycles. Thus, an originally large cylinder in the middle region is further expanded under the weak resultant force and then breaks into two independent cylinders. These two cylinders move towards two different walls, and their sizes decrease gradually until the smallest size is reached under the forceful interaction of the walls.

**Figure 7:**
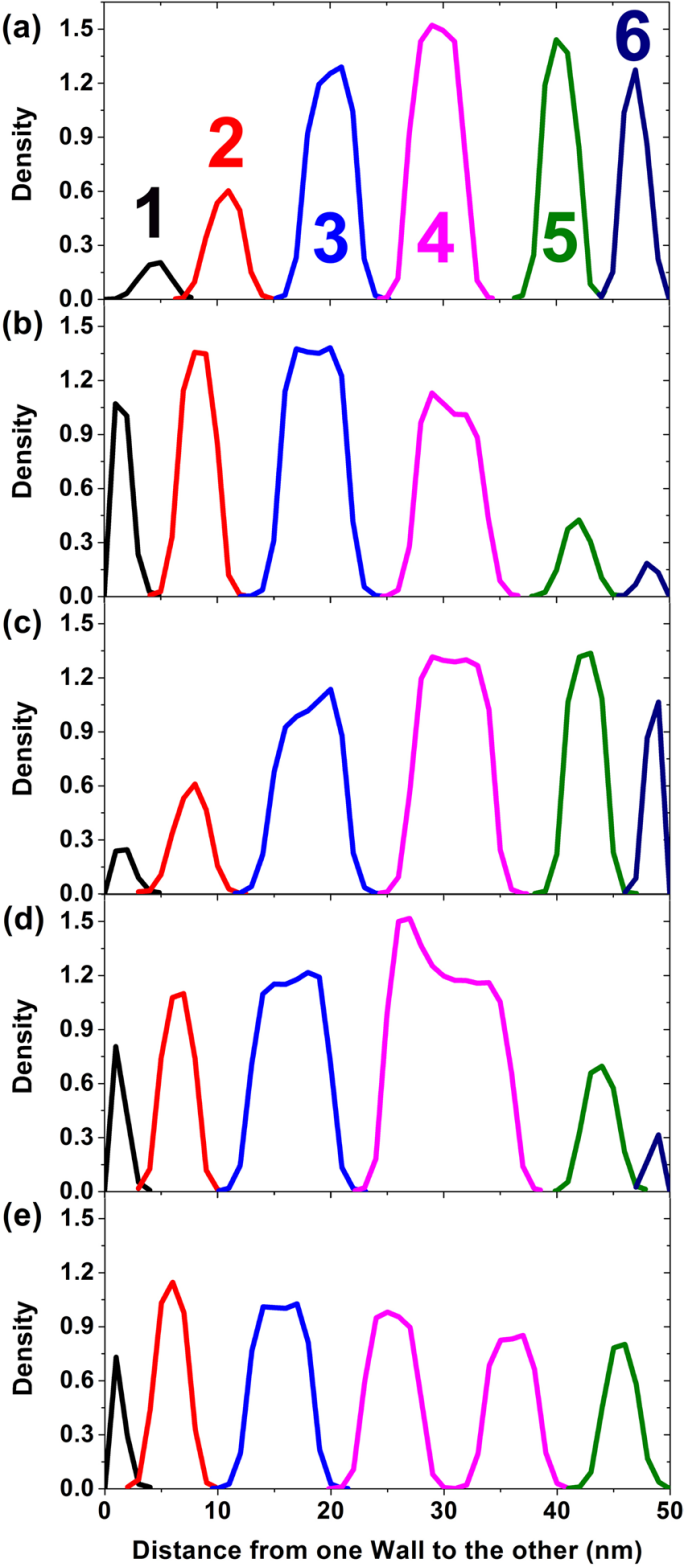
The density distribution of six aggregates in the vertical direction to the confinement at (**a**) 2 × 10^3^ steps, (**b**) 4 × 10^3^ steps, (**c**) 6 × 10^3^ steps, (**d**) 8 × 10^3^ steps and (**e**) 10 × 10^3^ steps.

The mechanism of YW55 confinement is illustrated by both the change in the density of the B beads in the six cylinders along the direction between the two walls and the change in the radial diameter, as shown in Fig. 7. As we know, the further away from the walls, the smaller force suffered, and thus, the densities of the cylinders along the Y axis, which is the acting direction of the repulsive force, present a gradient change at 2 × 10^3^ steps. After 2 × 10^3^ steps, a reversed trend occurred in the cylinders: the size of cylinders 1 and 6 decreased and the size of cylinders 3 and 4 increased, resulting from the transformation of the copolymer molecules in the cylinders. After another 2 × 10^3^ steps, the density nearly recovered to that at 2 × 10^3^ steps, under such “strong-weak-strong” periodic change in the repulsive force from one wall to the other. Accordingly, the volumes of cylinders 3 and 4 expanded further until reaching 8 × 10^3^ steps. In particular, for cylinder 4, an obvious high density and swollen volume were formed with the transformation of cylinders 5 and 6, and the resulting two-peak density indicates the formation of two cores. After an average distribution of compositions, cylinder 4 is split into two identical-sized cylinders with similar densities. Simultaneously, cylinder 6 merges into cylinder 5, resulting in the increased density of cylinder 5, which supplements the density decrease. At 10 × 10^3^ steps, the densities of the six cylinders become similar, and one cycle of the adjustment of both the density and size of the cylinders is finished.

## Conclusion

A symmetric coarse-grained copolymer, consisting of a stem with a hydrophobic component and two hydrophilic segments joined to each endpoint of the stem, was studied using MesoDyn simulations. Such copolymer molecules self-assemble into a cylindrical structure via layer-by-layer stacking of the self-assembled core-shell planar structure along the Z axis. These regular core-shell-structured cylinders are densely packed into a hexagonal arrangement. During the self-assembly process, the appearance of several vacancies accelerates the regulation of the cylinder size, especially when vacancies frequently arise in a short time, and the average size increases dramatically, indicating the beginning of the equilibrium stage, with only the regulation of the arrangement left. Judging from the occupied vacancies, the middle region is considered to be the inactive self-assembly region.

The effect of the two 1D confinements, i.e., the ZW55 and YW55 confinement, with the same interaction with both components of copolymer, on self-assembly were studied. The strong interaction environment resulting from ZW55 confinement, with both a short acting distance of 5 nm and large influencing area of 10,000 nm^3^, leads to free self-assembly along the Z axis. The copolymer molecules adopt different orientations to reduce the potential energy, and a core-shell micellar structure with hexagonal arrangement is formed. The introduction of such extensive confinement compresses the size of the micelles due to contraction of both the EO and MMA segments, and accordingly, the number of micelles increases. Vacancies seldom appear, and thus, the self-assembly mechanism under ZW55 confinement is only coalescence, which leads to a step-by-step decrease in the number of micelles. The cycled “contraction-expansion” change in the micellar size along the same route, combined with the corresponding change in the component distribution, help adjust the self-assembled arrangement. The long acting distance of 50 nm and small influencing area of 250 nm^3^ of YW55 confinement cannot change the self-assembly structure, so the cylindrical structure is maintained as it is in the thin film. However, an obvious classification of the cylinders with various sizes is realized under YW55 confinement. The largest-sized cylinders are generated in the middle region, owing to the lowest resultant force, leading the copolymers to adopt a more expanded conformation. Under the growth of the resultant force from both walls, large-sized cylinders break into two independent cylinders, which move towards the two walls; simultaneously, their sizes decrease until reaching the smallest sizes when they are closest to both walls. All results provide insight that can be utilized in the design and improvement of nanomaterials.
